# E-cigarette product preferences of Australian adolescent and adult users: a 2022 study

**DOI:** 10.1186/s12889-023-15142-8

**Published:** 2023-02-01

**Authors:** Michelle I Jongenelis

**Affiliations:** Melbourne Centre for Behaviour Change, Melbourne School of Psychological Sciences, University of Melbourne, 3010 Parkville, VIC Australia

**Keywords:** Electronic cigarettes, Nicotine, Flavouring, Preferences

## Abstract

**Background:**

Research that comprehensively documents preferences for different types of novel nicotine products in Australia is lacking, making it difficult for policymakers to determine where public health efforts should be focused. This study thus sought to explore Australian adolescent and adult e-cigarette users’ preferences for different types of e-cigarettes and e-liquids. Purchasing behaviours and sources of e-liquid were also examined.

**Methods:**

An online survey was administered to 4,617 Australians aged 12 + years, 636 of whom had used an e-cigarette in the last 30 days and were the focus of this study. Among users, 45% also smoked tobacco cigarettes, 41% were non-smokers, and 14% had never smoked.

**Results:**

The majority (82%) of e-cigarette users surveyed reported using nicotine-containing e-liquid in their devices. Fewer (60%) reported using non-nicotine e-liquid. The preference for nicotine over non-nicotine e-liquid was observed among all age and smoking groups. Most users (89%) reported using flavoured e-liquids, with adolescents (96%) significantly more likely than young adults (90%) and adults aged 25 + years (85%) to report using such e-liquids. Fruit flavours were the most popular among all subgroups. In terms of device type, disposable e-cigarettes were the most common product used among almost all groups; the exception being adults aged 25 + years who preferred systems with refillable tanks. Friends and tobacco retailers were the most frequently nominated sources of nicotine e-liquid among adolescents and young adults. Among adults aged 25 + years, tobacco retailers and the Internet were the most frequently nominated sources of these products.

**Conclusion:**

Disposable e-cigarettes with flavoured, nicotine-containing e-liquid are popular among users of the devices, including adolescents. Measures that restrict the accessibility and availability of flavoured e-liquids and disposable e-cigarettes, and greater enforcement of laws regarding the sale and importation of nicotine e-liquids, are urgently needed to protect youth and never smokers from these products.

**Supplementary Information:**

The online version contains supplementary material available at 10.1186/s12889-023-15142-8.

## Introduction

Recent years have seen significant global increases in the use of novel nicotine products, such as electronic cigarettes (e-cigarettes) [1]. In Australia, the context of the present study, figures from the National Drug Strategy Household Survey indicate an increase between 2016 and 2019 in current use of e-cigarettes among all age groups (adolescents and young adults: 3.7% in 2016 vs. 7.1% in 2019; adults: 1.2% in 2016 vs. 2.6% in 2019), with these increases observed among tobacco smokers and non-smokers [2].

The rapid growth in e-cigarette use is of concern to public health authorities [3, 4]. The e-liquids used in the devices contain toxicants that may be harmful to health [5–8], and there is considerable evidence linking e-cigarette use to short-term markers of possible health harms such as arterial stiffness, endothelial dysfunction, vascular oxidative stress, and decreased lung function capacity [9–13]. In a recent review documenting the risks associated with e-cigarette use, the addictive nature of nicotine (a chemical often found in e-liquids) was highlighted [14]. This review also found that non-smokers who use e-cigarettes are approximately three times more likely than those who avoid the devices to initiate cigarette smoking, raising concerns that e-cigarette use may contribute to a new population of smokers.

Multiple types of e-cigarettes and e-liquids are available on the market. E-cigarettes may be (i) pod-, cartridge-, or tank-based and/or (ii) refillable, reloadable, or disposable [15]. In terms of e-liquids, these are available in thousands of flavours and with or without nicotine. Information on the types of e-cigarettes and e-liquids preferred by Australian users of the devices is largely lacking as national surveys (e.g. National Drug Strategy Household Survey; Australian Secondary Students’ Alcohol and Drug Survey) must collect information on the use of a variety of drugs and are thus limited in their ability to comprehensively document preferences for different types of novel nicotine products. This makes it difficult for policymakers to determine where public health efforts should be focused.

Past research has attempted to address this knowledge gap, assessing product preferences among Australian young adult e-cigarette users [16]. This research found that 64% of e-cigarette users preferred nicotine-containing e-liquids and 89% preferred flavoured e-liquids. While this exploratory work provided much-needed information on the types of e-liquids young adults prefer to use, it did not identify the e-liquids Australians were actually using, nor did it assess use of various e-cigarette devices. This work was also limited by its focus on young adults to the exclusion of adolescents and other adults. Subsequent research conducted in Australian adults observed a preference for nicotine-containing e-liquid [17]. This research also assessed preferences for device type and found that box-tank e-cigarettes were the most popular devices among members of this population segment, followed by pen-tank e-cigarettes. Research examining the preferences of adolescents is lacking.

Although informative at the time they were published, these prior studies are outdated: the data reported were collected in 2016 and 2018. The e-cigarette market has grown considerably in recent years, with newer generation products now available. In addition, October 2021 saw Australia’s Therapeutic Goods Administration change the scheduling of nicotine, making the purchase of nicotine e-liquid or e-cigarettes that contain nicotine e-liquid illegal without a prescription, regardless of whether the intended use is for therapeutic purposes. Given these changes, an up-to-date examination of product preferences among Australian e-cigarette users is needed to appropriately inform control efforts.

The present study aimed to address the limited information on novel nicotine product use in Australia by exploring adolescent and adult e-cigarette users’ preferences for different types of e-cigarettes and e-liquids. Preferences were also examined by smoking status to determine the extent to which nicotine products are preferred by never smokers. A secondary aim of the study was to explore (i) users’ purchasing behaviours and (ii) sources of e-liquid. Results have the potential to inform the efforts of policymakers and public health agencies working to minimise uptake of these products.

## Method

An ISO-accredited web panel provider (Pureprofile) recruited and administered an online survey to a sample of 4,617 Australians aged 12 + years, of whom 14% (*n* = 636) were current users of e-cigarettes (reported using e-cigarettes of any type at least monthly and had used in the previous 30 days). Approval to conduct the study was obtained from the University of Melbourne’s Human Research Ethics Committee and all respondents provided informed consent. For those aged < 16 years, consent was also obtained from a parent/guardian. This involved the parent/guardian reading an online information sheet about the study and providing consent before being asked to leave the room so their child could provide consent and complete the survey in private. The study was conducted from the 1st to the 18th February 2022.

Respondents answered questions that assessed their gender, age, and socio-economic status (determined via postcode using the Australian Bureau of Statistics’ Socio-Economic Index for Areas ‘Index of Relative Disadvantage’ [18]). Respondents who reported being < 12 years of age were excluded from further participation.

E-cigarette user status was assessed by asking respondents whether they had ever used an e-cigarette, even just one or two puffs. Those who responded in the affirmative were then asked to report how often and on how many days in the last 30 days they used e-cigarettes (i) with nicotine, (ii) without nicotine, and (iii) with flavourings (response options presented in Table 1). Those who used an e-cigarette with nicotine were additionally asked to report on the strength of the e-liquid they usually used. Those who used an e-cigarette with flavourings were asked to report on the flavours they usually used, with responses to this open-ended question subsequently coded into Krusemann et al.’s [19] e-liquid flavour wheel categories. All users were asked to indicate the types of devices they usually used (e.g. disposable, pod-based, refillable; see Table 1 for all response options). Finally, all users were asked to report (i) whether they had purchased their own e-cigarette, only used those belonging to other people, or both and (ii) from where they usually obtained their nicotine and/or non-nicotine e-liquid (if applicable).

**Table 1:** Patterns of e-cigarette use, stratified by age and tobacco cigarette smoking status

	Overall	Age group			Smoking status^d^		
	%	Adolescent %	Young adult %	Adult 25+ %	Current smoker %	Non-smoker %	Never smoker %
Nicotine e-cigarette use^a^ Daily Weekly Monthly Less often than monthly Not at all now, but have used in the past Not at all now, and have never used Don’t know Strength usually used^ab^: 6 mg/ml 12 mg/ml 18 mg/ml 24 mg/ml Other Don’t know	*n* = 636 29 36 17 11 5 1 1 *n* = 521 42 27 6 2 6 17	*n* = 163 21 37 20 11 7 3 1 *n* = 127 43 17 6 2 6 26	*n* = 181 34 34 20 7 5 0 1 *n* = 157 36 22 6 3 9 24	*n* = 292 31 37 13 13 4 1 1 *n* = 237 45 35 6 2 4 8	*n* = 288 30 42 15 8 4 1 1 *n* = 250 38 32 9 3 5 13	*n* = 259 29 30 19 14 5 2 1 *n* = 202 47 20 3 1 5 24	*n* = 89 26 34 18 11 8 2 1 *n* = 69 44 28 4 1 10 13
Non-nicotine e-cigarette use^a^ Daily Weekly Monthly Less often than monthly Not at all now, but have used in the past Not at all now, and have never used Don’t know	*n* = 636 11 30 19 9 14 16 1	*n* = 163 8 30 20 6 18 18 < 1	*n* = 181 12 19 17 12 18 21 < 1	*n* = 292 13 36 20 9 9 13 < 1	*n* = 288 13 36 16 8 12 15 < 1	*n* = 259 10 23 22 11 17 16 1	*n* = 89 11 29 20 8 9 21 1
Flavoured e-cigarette use^a^ Daily Weekly Monthly Less often than monthly Not at all now, but have used in the past Not at all now, and have never used Flavour usually used^bc^: Fruit Menthol/Mint Other sweets Candy Other beverages Dessert Coffee/tea Tobacco Alcohol No preference Don’t know	*n* = 636 25 37 27 6 3 2 *n* = 565 64 11 6 5 4 2 2 2 1 3 2	*n* = 163 22 44 30 3 1 0 *n* = 156 65 9 6 4 7 1 1 0 1 7 1	*n* = 181 31 30 29 6 3 1 *n* = 162 80 3 1 8 5 1 1 1 0 1 2	*n* = 292 23 37 25 8 4 3 *n* = 247 54 18 9 4 1 3 4 3 2 2 3	*n* = 288 23 37 26 6 3 1 *n* = 249 57 12 9 6 2 1 3 2 1 3 4	*n* = 259 27 34 29 6 3 1 *n* = 232 71 10 4 6 5 4 2 1 1 3 0	*n* = 89 24 44 27 3 1 1 *n* = 84 67 11 2 2 5 0 2 1 0 6 4
Type of e-cigarette^c^ Disposable Pod-based Refillable tank Replaceable cartridges Mod system Don’t know	*n* = 636 52 37 36 21 12 3	*n* = 163 68 33 29 16 10 1	*n* = 181 64 41 35 22 8 3	*n* = 292 36 37 41 24 15 3	*n* = 288 43 41 40 26 16 3	*n* = 259 59 37 37 18 9 2	*n* = 89 62 27 24 16 6 2

*Note*. A figure of < 1 means the observed percentage was greater than 0 but less than 0.5

^a^Due to rounding, figures may not add to 100%

^b^Of those using at least monthly

^c^As multiple responses were permissible, proportions do not add to 100%

^d^Current smokers had smoked in the past 30 days and > 100 cigarettes in their lifetime; never smokers had never smoked a tobacco cigarette; non-smokers comprised all other respondents

Smoking status was assessed by asking respondents whether they had ever smoked a tobacco cigarette. Those responding in the affirmative were asked if they had smoked at least 100 cigarettes in their lifetime and on how many days they had smoked in the last 30 days. As per previous research [20], respondents were classified as current smokers if they reported smoking > 100 tobacco cigarettes in their lifetime and had smoked in the last 30 days. Respondents were classified as never smokers if they reported never smoking a tobacco cigarette. All other respondents were classified as non-smokers.

### Statistical analysis

As noted, only current users of e-cigarettes (defined as those who reported using e-cigarettes of any type at least monthly and had used in the previous 30 days) were of interest to the present study. Descriptive statistics were calculated for each of the following variables of interest: frequency of nicotine e-cigarette use, strength of nicotine e-liquid usually used, frequency of non-nicotine e-cigarette use, frequency of flavoured e-cigarette use, flavours of e-liquid usually used, types of e-cigarette device usually used, purchasing behaviour, and sources of nicotine and non-nicotine e-liquid. Statistics were calculated at the overall level, by age group (i.e. adolescents aged 12–17 years, young adults aged 18–24 years, and adults aged 25 + years), and by smoking status (current smoker, non-smoker, never smoker). Results for additional age groups (25–29 years, 30–39 years, 40–49 years, 50–59 years, 60–69 years, and 70 + years) are presented in the online supplementary material (Tables S1 and S2).

Pearson chi-square tests were conducted to examine differences by age group and smoking status for the following variables: (i) frequency of nicotine, non-nicotine, and flavoured e-cigarette use; (ii) type of e-cigarette device used; (iii) purchasing behaviour; and (iv) source of nicotine e-liquid. Missing data were treated listwise.

## Results

### Sample

The demographic profile of the sample is presented in Table 2. For gender and smoking status, the profile of current e-cigarette users sampled was found to be consistent with data collected nationally and internationally [2, 21, 22].

**Table 2:** Sample profile (n = 636)

Demographic characteristic	*n*	%	
Gender Man Woman	348 288	55 45	
Age 12 to 17 years 18 to 24 years 25 + years	163 181 292	26 28 46	
Range (in years)	12–70		
Socio-economic status^a^ Low (deciles 1–4) Mid (deciles 5–8) High (deciles 9–10) Missing	228 272 130 6	36 43 20 1	
Smoking status^b^ Current smoker Non-smoker Never smoker	288 259 89	45 41 14	

^a^As per Australian Bureau of Statistics’ Socio- Economic Index for Areas ‘Index of Relative Disadvantage’

^b^Current smokers had smoked a tobacco cigarette in the past 30 days and > 100 cigarettes in their lifetime; never smokers had never smoked a tobacco cigarette; non-smokers comprised all other respondents

Among current users of e-cigarettes, 82% used nicotine-containing e-liquids and 60% used non-nicotine e-liquids at least monthly (Table 1). Nearly half (47%) used both nicotine and non-nicotine e-liquids at least monthly. A preference for nicotine over non-nicotine e-liquid was observed among all age groups (adolescents: 78% using nicotine e-liquid at least monthly cf. 58% using non-nicotine e-liquid at least monthly; young adults: 87% cf. 49%; adults aged 25 + years: 81% cf. 69%). A preference for nicotine over non-nicotine e-liquid was also observed among current smokers (87% using nicotine e-liquid at least monthly cf. 65% using non-nicotine e-liquid at least monthly), non-smokers (78% cf. 54%), and never smokers (78% cf. 61%). Of those using nicotine e-liquid at least monthly, 6 mg/ml was the most common nicotine strength used among all subgroups, followed by 12 mg/ml. A quarter of adolescent and young adult nicotine e-liquid users reported that they did not know the nicotine strength of the e-liquid they used.

Pearson chi-square tests showed no differences between adolescents, young adults, and adults aged 25 + years for ‘at least monthly’ nicotine e-liquid use (all *p*s > 0.05). A significant age difference was observed for non-nicotine e-liquid use, with adults more likely than adolescents (*p* = .019, φ = 0.11) and young adults (*p* < .001, φ = 0.20) to report using this type of e-liquid at least monthly. For smoking status, current smokers were significantly more likely than never smokers (*p* = .045, φ = 0.10) and non-smokers (*p* = .008, φ = 0.11) to report using nicotine e-liquid. Current smokers were also more likely than non-smokers to report using non-nicotine e-liquid (*p* = .014, φ = 0.11).

The vast majority (89%) of current e-cigarette users reported using a flavoured e-liquid at least monthly (adolescents: 96%, young adults: 90%, adults aged 25 + years: 85%; current smokers: 87%, non-smokers: 90%, never smokers: 94%), with fruit flavoured e-liquids the most used among all subgroups. Adolescents were significantly more likely than young adults (*p* = .030, φ = 0.12) and adults (*p* < .001, φ = 0.17) to report using flavoured e-liquids at least monthly. For smoking status, never smokers were significantly more likely than current smokers to report using flavoured e-liquids at least monthly (*p* = .042, φ = 0.11).

In terms of device type, disposable e-cigarettes were the most used among almost all groups; the exception being adults aged 25 + years who preferred systems with refillable tanks. Pod-based e-cigarettes were also popular, with around one-third of respondents reporting use of these devices. Several age differences were identified. Adolescents (*p* < .001, φ = 0.31) and young adults (*p* < .001, φ = 0.27) were significantly more likely than adults aged 25 + years to report using disposable e-cigarettes. Adults aged 25 + years were significantly more likely than adolescents to report using devices with refillable tanks (*p* = .008, φ = 0.13) and replaceable cartridges (*p* = .037, φ = 0.10). Adults aged 25 + years were more likely than young adults to report using mod systems (*p* = .038, φ = 0.10). For smoking status, never smokers (*p* = .002, φ = 0.16) and non-smokers (*p* < .001, φ = 0.16) were more likely than current smokers to use disposable e-cigarettes. Current smokers were more likely than never smokers to use pod-based devices (*p* = .020, φ = 0.12), devices with refillable tanks (*p* = .005, φ = 0.14), devices with replaceable cartridges (*p* = .039, φ = 0.11), and mod systems (*p* = .015, φ = 0.13). Current smokers were more likely than non-smokers to use replaceable cartridges (*p* = .016, φ = 0.10) and mod systems (*p* = .025, φ = 0.10). Non-smokers were more likely than never smokers to use refillable tanks (*p* = .024, φ = 0.12).

For purchasing behaviours (Table 3), just over half (56%) of current users reported purchasing their own e-cigarette, and one-quarter (26%) reported only using devices that belonged to others. Results varied by age group, with nearly three-quarters (71%) of adults aged 25 + years purchasing their own e-cigarettes compared to 35% of adolescents (*p* < .001, φ = 0.35) and 51% of young adults (*p* < .001, φ = 0.19). Young adults were also more likely than adolescents to report purchasing their own device (*p* = .002, φ = 0.17). For smoking status, current smokers were significantly more likely than never smokers (*p* = .009, φ = 0.14) and non-smokers (*p* < .001, φ = 0.14) to report purchasing their own device.

**Table 3:** *Purchasing behaviours and sources of e-liquids, stratified by age and tobacco cigarette smoking status*

	Overall	Age group			Smoking status^c^		
	%	Adolescent %	Young adult %	Adult 25+ %	Current smoker %	Non-smoker %	Never smoker %
Purchasing behaviour^a^ Has purchased own e-cigarette Only used other people’s e-cigarettes Both	*n* = 636 56 26 18	*n* = 163 35 41 24	*n* = 181 51 25 24	*n* = 292 71 17 12	*n* = 288 64 20 16	*n* = 259 50 28 22	*n* = 89 48 38 14
Source of nicotine e-liquid Internet Friend Family member Smoke shop, tobacco specialty store or outlet Specialised store selling vaping devices and liquids (not online) Petrol station Convenience store Pharmacy/chemist Other Don’t know/Can’t say	*n* = 623 21 27 4 28 10 1 5 1 1 2	*n* = 157 8 52 5 20 5 1 3 1 1 4	*n* = 180 16 27 4 30 13 2 6 1 0 2	*n* = 286 31 13 3 31 11 2 5 2 1 2	*n* = 284 25 18 6 30 12 1 4 2 1 2	*n* = 253 18 32 2 29 10 1 5 < 1 < 1 2	*n* = 86 13 42 3 17 5 5 8 4 0 3
Source of non-nicotine e-liquid Internet Friend Family member Smoke shop, tobacco specialty store or outlet Specialised store selling vaping devices and liquids (not online) Petrol station Convenience store Pharmacy/chemist Other Don’t know/Can’t say	*n* = 527 20 27 3 25 11 1 3 2 < 1 7	*n* = 132 10 46 4 15 8 1 3 1 1 11	*n* = 142 15 30 6 25 9 1 4 3 0 7	*n* = 253 29 16 2 30 14 1 3 1 0 4	*n* = 243 22 21 4 28 14 1 3 1 1 5	*n* = 215 17 29 3 26 9 1 3 2 0 9	*n* = 69 22 41 4 13 9 0 4 1 0 6
Source of e-liquid^b^ Internet Friend Family member Smoke shop, tobacco specialty store or outlet Specialised store selling vaping devices and liquids (not online) Petrol station Convenience store Pharmacy/chemist Other Don’t know/Can’t say	*n* = 4 0 75 25 0 0 0 0 0 0 0	*n* = 1 0 100 0 0 0 0 0 0 0 0	*n* = 1 0 100 0 0 0 0 0 0 0 0	*n* = 2 0 50 50 0 0 0 0 0 0 0	*n* = 1 0 100 0 0 0 0 0 0 0 0	*n* = 2 0 100 0 0 0 0 0 0 0 0	*n* = 1 0 0 100 0 0 0 0 0 0 0

^a^Due to rounding, figures may not add to 100%

^b^Among those who reported that they did not know if the e-liquid they used contained nicotine

^c^Current smokers had smoked in the past 30 days and > 100 cigarettes in their lifetime; never smokers had never smoked a tobacco cigarette; non-smokers comprised all other respondents

Friends were the most common source of both nicotine and non-nicotine e-liquid for adolescents, followed by tobacco retailers. Among young adults, tobacco retailers were the most common source of nicotine e-liquid followed by friends, whereas the reverse was true for non-nicotine e-liquid. The Internet and tobacco retailers were the most frequently nominated sources of nicotine and non-nicotine e-liquid among adults aged 25 + years. Several age differences were identified. Adolescents were significantly more likely than young adults and adults aged 25 + years to report sourcing their nicotine e-liquid from friends (both *p*s < 0.001) and significantly less likely to report sourcing from the Internet (cf. young adults: *p* = .018; cf. adults: *p* < .001), tobacco retailers (cf. young adults: *p* = .043; cf. adults: *p* = .019), or a specialised vape store (cf. young adults: *p* = .015; cf. adults: *p* = .032). Young adults were significantly more likely than adults to report sourcing their nicotine e-liquid from a friend (*p* < .001) and significantly less likely to report sourcing from the Internet (*p* < .001).

Several differences by smoking status were identified. Current smokers were significantly more likely than never smokers to report purchasing their e-liquid from the Internet (*p* = .014) and tobacco retailers (*p* = .022) whereas never smokers were more likely than current smokers to report sourcing from friends (*p* < .001) and petrol stations (*p* = .032). Current smokers were more likely than non-smokers to report purchasing their e-liquid from the Internet (*p* = .045) and family members (*p* = .013) but less likely to report purchasing from friends (*p* < .001). Non-smokers were significantly more likely than never smokers to report sourcing their nicotine e-liquid from tobacco retailers (*p* = .032) whereas never smokers were more likely than non-smokers to report sourcing from petrol stations (*p* = .019).

## Discussion

To assist policymakers and public health agencies determine where public health measures addressing rising rates of e-cigarette use should be directed, the present study assessed e-cigarette product preferences in a sample of adolescent and adult users of the devices. A particularly notable contribution of the present study was the sampling of Australian adolescents, a population in whom e-cigarette product preferences do not appear to have been examined.

A preference for nicotine-containing, flavoured e-liquids was observed among all groups, and almost all groups exhibited a preference for disposable and pod-based devices. The findings of this study have several implications for public health. First, the use of nicotine e-liquids by the majority of adolescent and young adult e-cigarette users surveyed is concerning, as is the finding that a quarter of adolescent and young adult nicotine users did not know the strength of the nicotine e-liquid they used. Given the potential risks associated with nicotine exposure in adolescence and young adulthood [23], these results indicate that most e-cigarette users within these population groups are at considerable risk of harm.

Second, the use of nicotine reported by adolescents and never smokers in the present study supports evidence that these products are being sold in Australia illegally [24]. Australia has placed restrictions on nicotine-containing e-liquid such that the sale of these liquids outside the pharmaceutical model is prohibited and those wishing to use these liquids are required to obtain a prescription from a registered health practitioner with whom they have spoken about smoking cessation [25]. Given never smokers are not using e-cigarettes to quit smoking, and very few adolescents report using e-cigarette products for smoking cessation purposes [2], it stands to reason that members of these groups are sourcing nicotine e-liquid unlawfully. Indeed, a quarter of adolescents reported sourcing their nicotine e-liquid from tobacco or vaping retailers, despite it being illegal to sell these products to minors. One-fifth of never smokers surveyed also reported sourcing nicotine e-liquid from these stores, likely doing so without a prescription. Greater enforcement of laws regarding the sale of liquid nicotine and closure of the Therapeutic Goods Administration’s Personal Importation Scheme (which allows importation of nicotine e-liquid from overseas) are potential means of addressing the use of nicotine e-liquid among those who are not using the product for smoking cessation purposes. In terms of the former, enforcement is currently hampered by the absence of positive licensing schemes in the states of New South Wales, Queensland, and Victoria. The introduction of such schemes is critical to facilitate monitoring of retailer compliance and optimise enforcement of existing laws.

Third, consistent with prior Australian research conducted on young adults [16], the vast majority of users in all groups surveyed used flavoured e-liquids, with fruit flavours preferred. Adolescents were significantly more likely than young adults and adults to report using flavoured e-liquids at least monthly, supporting previous research that found these products to be appealing to youth [26]. Results also showed that never smokers were significantly more likely than current smokers to report using flavoured e-liquids at least monthly, suggesting that such e-liquids facilitate recreational use of vaping products. Given (i) the appeal of flavoured e-liquids among youth and never smokers and (ii) evidence indicating that flavourings increase the palatability of e-cigarettes [27], prohibiting flavoured e-liquids has the potential to reduce the attractiveness of use among all users, especially those using recreationally.

Finally, results demonstrate the popularity of disposable and pod-based e-cigarettes, especially among adolescents, young adults, and never smokers. Such products are cheaper than other types of e-cigarettes [28, 29], and it has been suggested that their inexpensiveness is a potential risk factor for youth uptake [30]. Of further concern, the e-liquids in these types of e-cigarettes are typically nicotine-salt-based. The lower pH of these e-liquids reduces the harshness of the inhaled aerosol, making the e-liquid highly palatable and easy to inhale [31, 32] and resulting in more intense puffing and greater nicotine delivery [33]. The preference for these high-strength devices among the adolescents, young adults, and never smokers surveyed is thus concerning and efforts should be made to reduce the availability of these products to minimise the risk of addiction from recreational e-cigarette use.

The aforementioned findings should be interpreted in light of this study’s limitations. First, recruitment was via an online panel provider, which limits the generalisability of the results. Second, it is not possible to determine sample representativeness as available national surveys do not report the demographic characteristics of current e-cigarette users. However, the profile of users in the present sample is consistent with data collected nationally and internationally in terms of gender and smoking status [2, 21, 22]. Finally, whether e-cigarette product preferences change with age could not be assessed as data were collected at a single point in time. Accordingly, it cannot be determined if the preference for disposable and pod-based devices observed among adolescents and young adults persists or if these users migrate to using more sophisticated refillable tanks, which was the preferred device among those aged 25 + years. Prospective cohort studies that explore the trajectory of e-cigarette use among users over time may provide a greater understanding of changes in product preferences, and thus have the potential to further inform policy and practice.

In conclusion, the present study provides useful data on e-cigarette product preferences that are absent from national surveys and that can be used to inform efforts to halt rising rates of e-cigarette use among youth and never smokers in Australia. Measures that restrict the accessibility and availability of flavoured e-liquids and disposable e-cigarettes, and greater enforcement of laws regarding the sale of nicotine-containing e-liquids, are urgently needed.

## Electronic supplementary material

Below is the link to the electronic supplementary material.

Supplementary Material 1

## Data Availability

The dataset analysed during the current study is available from the corresponding author on reasonable request.
