# Supplementary material for: E-cigarette product preferences of Australian adolescent and adult users: a 2022 study
Source: BMC Public Health. 2023 Feb 1;23:220. doi: 10.1186/s12889-023-15142-8 (PMC9893577; doi:10.1186/s12889-023-15142-8)
Supplement: Supplementary file 1 — Supplementary Material 1 [file 12889_2023_15142_MOESM1_ESM.docx]

**Supplementary Material**

Table S1.

*Patterns of e-cigarette use, stratified by further age categories*

|  | Age group (in years) | | | | | |
| --- | --- | --- | --- | --- | --- | --- |
|  | 25 – 29  % | 30 – 39  % | 40 – 49  % | 50 – 59  % | 60 – 69  % | 70+  % |
| Nicotine e-cigarette use^a^  Daily  Weekly  Monthly  Less often than monthly  Not at all now, but have used in the past  Not at all now, and have never used  Don’t know  Strength usually used^ab^:  6mg/ml  12mg/ml  18mg/ml  24mg/ml  Other  Don’t know | *n* = 40  13  43  17  22  5  0  0  *n* = 29  52  24  3  0  0  21 | *n* = 126  31  40  13  9  4  2  1  *n* = 106  2  46  34  9  2  7 | *n* = 73  27  40  12  15  4  1  0  *n* = 58  45  36  3  2  9  5 | *n* = 32  50  25  13  9  3  0  0  *n* = 28  32  54  4  7  0  4 | *n* = 19  42  21  16  16  0  0  5  *n* = 15  7  40  27  7  7  13 | *n* = 2  50  0  0  50  0  0  0  *n* = 1  100  0  0  0  0  0 |
| Non-nicotine e-cigarette use^a^  Daily  Weekly  Monthly  Less often than monthly  Not at all now, but have used in the past  Not at all now, and have never used  Don’t know | *n* = 40  10  30  35  5  5  15  0 | *n* = 125  16  40  18  10  9  8  0 | *n* = 73  12  45  18  14  1  10  0 | *n* = 32  13  19  12  6  22  28  0 | *n* = 18  6  22  22  6  22  22  0 | *n* = 2  0  0  0  0  1  1  0 |
| Flavoured e-cigarette use^a^  Daily  Weekly  Monthly  Less often than monthly  Not at all now, but have used in the past  Not at all now, and have never used  Flavour usually used^bc^:  Fruit  Menthol/Mint  Other sweets  Candy  Other beverages  Dessert  Coffee/tea  Tobacco  Alcohol  No preference  Don’t know | *n* = 40  8  35  32  15  5  5  *n* = 30  53  13  3  7  0  0  0  0  3  10  0 | *n* = 126  26  36  27  6  2  3  *n* = 113  54  22  8  3  2  4  3  3  0  1  4 | *n* = 73  23  41  23  7  6  0  *n* = 64  63  14  11  5  0  6  5  2  2  3  0 | *n* = 32  31  34  19  3  6  6  *n* = 27  41  11  15  4  0  0  11  7  7  0  7 | *n* = 19  16  32  16  16  10  10  *n* = 12  33  25  17  8  0  0  0  17  0  0  0 | *n* = 2  0  0  1  0  1  0  *n* = 1  100  0  0  0  0  0  0  0  0  0  0 |
| Type of e-cigarette^c^  Disposable  Pod-based  Refillable tank  Replaceable cartridges  Mod system  Don’t know | *n* = 40  45  43  25  20  10  10 | *n* = 126  37  44  42  26  13  2 | *n* = 73  40  36  44  18  19  3 | *n* = 32  28  25  47  28  16  3 | *n* = 19  11  11  47  42  21  5 | *n* = 2  0  0  100  0  0  0 |

^a^Due to rounding, figures may not add to 100%.

^b^Of those using at least monthly.

^c^As multiple responses were permissible, proportions do not add to 100%.

Table S2.

*Purchasing behaviours and sources of e-liquids, stratified by further age categories*

|  | Age group (in years) | | | | | |
| --- | --- | --- | --- | --- | --- | --- |
|  | 25 – 29  % | 30 – 39  % | 40 – 49  % | 50 – 59  % | 60 – 69  % | 70+  % |
| Purchasing behaviour^a^  Has purchased own e-cigarette  Only used other people’s e-cigarettes  Both | *n* = 40  50  28  23 | *n* = 126  71  15  14 | *n* = 73  80  15  5 | *n* = 32  69  19  12 | *n* = 19  79  16  25 | *n* = 2  50  50  0 |
| Source of nicotine e-liquid^a^  Internet  Friend  Family member  Smoke shop, tobacco specialty store or outlet  Specialised store selling vaping devices and liquids (not online)  Petrol station  Convenience store  Pharmacy/chemist  Other  Don’t know/Can’t say | *n* = 40  25  20  5  30  5  0  3  5  0  7 | *n* = 122  32  12  3  35  7  3  5  3  0  0 | *n* = 72  22  14  1  33  15  0  8  0  3  3 | *n* = 32  41  9  0  25  13  3  6  3  0  0 | *n* = 18  50  6  6  6  33  0  0  0  0  0 | *n* = 2  50  50  0  0  0  0  0  0  0  0 |
| Source of non-nicotine e-liquid^a^  Internet  Friend  Family member  Smoke shop, tobacco specialty store or outlet  Specialised store selling vaping devices and liquids (not online)  Petrol station  Convenience store  Pharmacy/chemist  Other  Don’t know/Can’t say | *n* = 34  15  26  3  26  15  3  3  3  0  6 | *n* = 115  34  13  2  30  14  1  3  1  0  3 | *n* = 66  23  17  3  32  15  0  4  0  0  6 | *n* = 23  30  17  0  44  4  4  0  0  0  0 | *n* = 14  0  0  100  0  0  0  0  0  0  0 | *n* = 1  100  0  0  0  0  0  0  0  0  0 |
| Source of e-liquid^ab^  Internet  Friend  Family member  Smoke shop, tobacco specialty store or outlet  Specialised store selling vaping devices and liquids (not online)  Petrol station  Convenience store  Pharmacy/chemist  Other  Don’t know/Can’t say | *n* = 0  -  -  -  -  -  -  -  -  -  - | *n* = 1  0  100  0  0  0  0  0  0  0  0 | *n* = 0  -  -  -  -  -  -  -  -  -  - | *n* = 0  -  -  -  -  -  -  -  -  -  - | *n* = 1  0  100  0  0  0  0  0  0  0  0 | *n* = 0  -  -  -  -  -  -  -  -  -  - |

^a^Due to rounding, figures may not add to 100%.

^b^Among those who reported that they did not know if the e-liquid they used contained nicotine.
